# Toward an Integrative Approach for Making Sense Distinctions

**DOI:** 10.3389/frai.2022.745626

**Published:** 2022-02-07

**Authors:** John P. McCrae, Theodorus Fransen, Sina Ahmadi, Paul Buitelaar, Koustava Goswami

**Affiliations:** Data Science Institute, National University of Ireland Galway, Galway, Ireland

**Keywords:** lexicography, word senses, semantics, distributional semantics, cognitive semantics, multilinguality, generative lexicon, wordnets

## Abstract

Word senses are the fundamental unit of description in lexicography, yet it is rarely the case that different dictionaries reach any agreement on the number and definition of senses in a language. With the recent rise in natural language processing and other computational approaches there is an increasing demand for quantitatively validated sense catalogues of words, yet no consensus methodology exists. In this paper, we look at four main approaches to making sense distinctions: formal, cognitive, distributional, and intercultural and examine the strengths and weaknesses of each approach. We then consider how these may be combined into a single sound methodology. We illustrate this by examining two English words, “wing” and “fish,” using existing resources for each of these four approaches and illustrate the weaknesses of each. We then look at the impact of such an integrated method and provide some future perspectives on the research that is necessary to reach a principled method for making sense distinctions.

## 1. Introduction

Although word senses are the most fundamental unit of meaning, there is no widely-agreed definition of what a sense is. In fact, some lexicographers have even claimed that word senses do not exist (Kilgarriff, 1997). Nonetheless, word senses are the basic organizational paradigm of a dictionary and are vitally important for a number of other applications in natural language processing (NLP), most notably word sense disambiguation. As such, the modern lexicographer is left with little more than very loose ideas of how to define word senses, based on their intuition and existing lexicographic practices such as copying from other dictionaries. This leaves us without a clear explanation for why sense distinctions are made that can provide any insights into the cognitive and semantic processes that underlie word senses.

Currently, there are several approaches to word senses based on very different domains of lexical semantics. Firstly, formal approaches to lexical semantics define senses by expressions in a logical or written form. The formal approach very naturally generates senses, but can often lead to splitting the senses into many very specific senses, such as seen in WordNet (Miller, 1995; Fellbaum, 2010). To avoid this, formal theories such as the generative lexicon (Pustejovsky, 1991) have attempted to group senses into regular classes despite the lack of large-scale practical evaluation of such theories. Secondly, in cognitive approaches, word meaning is connected to meaning through evidence including fMRIs and cognitive experiments. In particular, there has been some study on the use of word association norms (De Deyne et al., 2019) leading to the recently released Small World of Words Lexicon^1^. A third approach is based on the distributional hypothesis—“you shall know a word by the company it keeps”—which is currently proving extremely successful in NLP applications driven by large computational models such as word2vec (Mikolov et al., 2013).

Although distributional models mostly do not model senses directly, recent “contextualized” models such as BERT introduce a computational idea of a sense by inferring a distinct vector for each occurrence of a word, rather than having a single vector for all senses of a word. Moreover, injecting external knowledge (e.g., sense definition) into contextualized models enhances efficiency of distributional models. While distributional models have both theoretical limitations (Pustejovsky and Jezek, 2008) and uninspiring practical results (Nair et al., 2020), the rapid progress of these models is driving results, and in combination with other theories may lead to breakthroughs.

Finally, senses can be understood from a cross-cultural perspective, by which we mean leveraging the large amount of parallel data from different languages based on the intuition that word senses are distinct if they are translated differently. By combining data from a large number of languages, including minority and historical languages, senses can be efficiently inferred.

In this paper, we discuss the different methods of making senses and how these could be practically applied by a working lexicographer. We take as examples two common English nouns, “fish” and “wing,” which exemplify the challenges we have in making distinctions between senses, and as common nouns with a concrete primary sense represent an average challenge for lexicographers in having many senses but also clearly defined meanings. We use these examples to highlight the challenges that have been raised in the literature and to illustrate the difficulties that exist in combining multiple approaches.

The rest of the paper is structured as follows: in section 2 we discuss some of the background and definitions that have been used for making sense distinctions. Then in section 3, we discuss polysemy from the four perspectives already seen and show how a working lexicographer can make sense distinctions using quantitative and qualitative methods. We then apply this in section 4 to the two words “fish” and “wing” and evaluate the approaches on these words. Finally, in section 5, we consider the potential impact of a method for making sense distinctions and how it may be achieved, before concluding in section 6.

## 2. Background

Currently, there is no clear methodology for distinguishing senses in a dictionary that can be used in practice by lexicographers and this has led to the conclusion that the problem is insoluble (Lyons and John, 1995; Kilgarriff, 1997). Further, the conclusions of experiments with word sense disambiguation have shown that fine-grained sense catalogues, such as WordNet (Miller, 1995; Fellbaum, 2010), have poor inter-annotator agreement (Snyder and Palmer, 2004), and have led to the development of more coarse-grained catalogues (Hovy et al., 2006; Navigli, 2006; Snow et al., 2007; Dandala et al., 2013). Yet, acquiescing to the problem would miss the many subtle distinctions made in a language and fail in the basic role of a lexicographer to document the language.

One of the main approaches to formal sense distinctions has been through the idea of the generative lexicon (Pustejovsky, 1991), where words are understood in terms of four main aspects: formal (what is it?), telic (what does it do?), constitutive (what is it made of?) and agentative (what is it used for?). This theory has not gained much traction in the lexicographic community, with the only dictionary-like resource created on these principles being CORELEX (Buitelaar, 1998). This approach also leads to a form of “underspecification,” where new senses are created by a process of “coercion” from a core meaning, but this has been criticized due to the difficulty in finding a “common core that encompasses [all] different senses” (Vicente, 2018). An alternative model of “overspecification” where there is a long list of (potentially overlapping) senses, from which one has been selected, can be disputed by examples of new senses being formed by speakers on-the-fly through processes such as metonymy or metaphor (e.g., “handbag” in Kilgarriff, 1997). Such a method would also likely be too onerous for almost all lexicographers.

With the rise of modern NLP, an increasing focus has been made on distributional methods. While the first methods simply ignored word senses by finding the representation of words as lemmata as in word2vec (Mikolov et al., 2013), more recent models such as BERT (Devlin et al., 2019) and SensEmBERT (Scarlini et al., 2020) induce a distinct representation of a word for each context. In effect, this creates a distinct sense for every occurrence of a word, and this has even been transformed into a context-specific definition system, Generationary (Bevilacqua et al., 2020), thus creating a maximally over-specified dictionary with a definition for every single occurrence. In addition, it has been shown that the use of a formal sense inventory such as WordNet can improve performance on NLP tasks (Rothe and Schütze, 2015; Levine et al., 2020).

That being said, several limitations have been observed with distributional approaches, including their inability to “explain the rich variation in linguistic meaning in language” (Pustejovsky and Jezek, 2008) or to model linguistic phenomena such as entailment (Westera and Boleda, 2019). Distributional methods are effective at distinguishing between homonymous words (Lake and Murphy, 2020) but show much poor performance for subtle polysemy distinctions (Nair et al., 2020).

In order to establish external evidence for word sense distinctions, inter-linguistic comparison has often been used based on the hypothesis that the inventory of senses are shared between languages, but may be assigned to different words. This hypothesis has been well-validated for homonymy, leading to the “One Homonym per Translation” hypothesis (Hauer and Kondrak, 2020). This has been robustly evaluated for “type-A homonyms” where two senses of a word are caused by two words of different etymologies developing a single form. Further, this has proved to be an effective method both for sense tagging (Diab and Resnik, 2002) and for constructing sense catalogues (Bansal et al., 2012).

Cognitive experiments can provide direct evidence for word sense distinctions, and fMRI studies have shown that there is real evidence for distinctions between homonyms (Copland et al., 2007). More evidence can be extracted through word association experiments to develop Word Association Norms (WANs), as in the recently published “Small World of Words” dataset (De Deyne et al., 2019). WANs are easily collected in human experiments and have been shown to be similar to sense distinctions in traditional dictionaries (Reyes-Magaña et al., 2020).

These theoretical perspectives have so far examined only part of the problem of making sense distinctions and vary from being completely quantitative to completely theoretical. There have not been many studies looking across theories and combining quantitative and qualitative research methodologies (Branco et al., 2020).

## 3. Defining Polysemy

**Figure 1:**
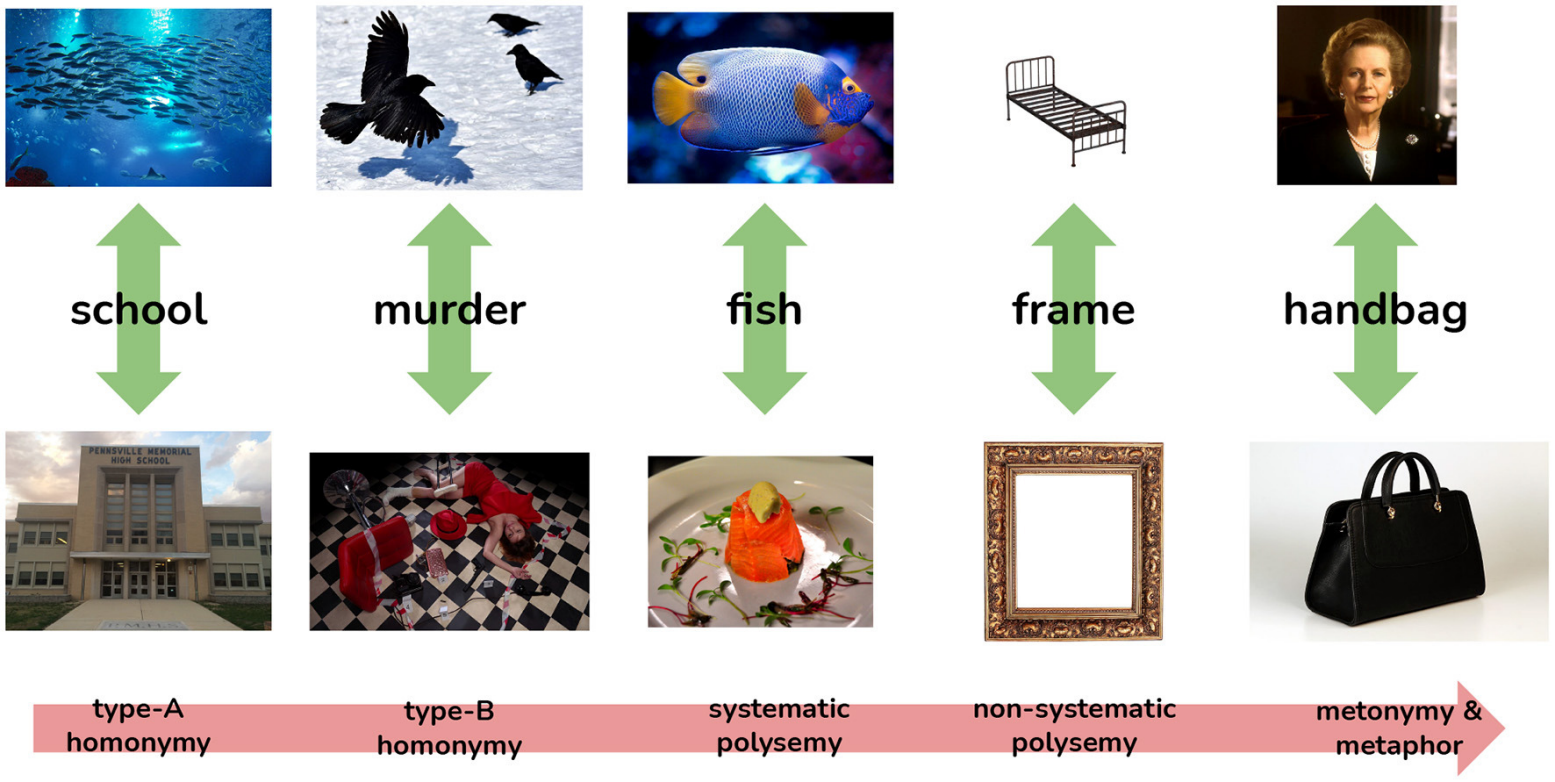
The spectrum of polysemy, representing how certain kinds of sense distinctions correspond to the ease and agreement with which these distinctions are made by lexicographers.

Polysemy can be viewed as a spectrum and we distinguish five principal areas on this spectrum as depicted in Figure 1. This spectrum represents word sense by their perceived distinctness with homonyms that are distinguished by multiple criteria, universally distinguished in dictionaries and easily distinguished by automatic methods at one end and conversely word senses at the other end that are scarcely distinguished by lexicographers or automatic methods. The ordering of these categories is based on the authors' experience and it is an open question if this scale corresponds to the accuracy of the methods discussed in this paper. Homonymy refers to multiple meanings of a word that are not semantically related and are further distinguished into “type-A” and “type-B” (Hauer and Kondrak, 2020), where type-A homonyms have distinct etymology, for example a “school of fish” (from Middle Dutch “skole,” related to “shoal”) vs. “high school” (from Greek “$\sigma \chi o\lambda \overset{´}{\eta }$” via Latin “schola”). In contrast, a “murder of crows” is a quite distinct meaning from the crime but is an extension of the meaning of the original word. For polysemy, we distinguish between those that can be understood systematically, e.g., “fish” as an animal vs. “fish” as a meat, in contrast to “bed frame” vs. “picture frame.” Finally, we have the case where words may take specific meanings in context, such as Kilgarriff (1997)'s analysis of “handbag” referring to the UK prime minister, Margaret Thatcher. In lexicography, the case of homonymy is generally well handled and all formal, cognitive, distributional, and cross-cultural methods are able to distinguish these senses with high accuracy. Similarly, metonymy^2^ is rarely of interest to lexicographers as such senses tend to be *hapax legomenon* phenomena, which only occur once in a corpus and are not useful to provide to users of the dictionary. As such, the main interest here is in polysemy rather than homonymy or metonymy, but the boundaries of these are not always clear.

In a case study on the definitions provided across four dictionaries for the polysemous English word “crawl,” Fillmore and Atkins (2000) illustrated the problematic nature of dictionary-induced sense alignment. “The underlying contention is that many of the difficulties experienced by current treatments of polysemy do not spring from the nature of polysemy itself, but from more general problems of semantic and lexicographic methodology, in particular the lack of a clear, practical, and verifiable technique for framing lexical definitions” (Goddard, 2000).

In this paper, we will survey and describe the methodologies that have been applied to the task of defining word senses in the lexicon and, in particular, focus on the interaction between the different theories. We do not focus on homonyms as it has already been established that these methods are easily distinguishable by a range of methods (Copland et al., 2007; Branco et al., 2020; Hauer and Kondrak, 2020; Nair et al., 2020). We will then complete this study with a number of practical examples drawn both from practical experience with developing an open-source lexicographic resource (McCrae et al., 2019) as well as by studying the sense distinctions made in existing dictionaries and how they can be explained on the four axes of formal, cognitive, distributional, and cross-cultural lexical semantics and lay out the basis of an integrative approach that uses evidence from all approaches.

In order to provide lexicographers with a practical methodology for creating sense inventories given these blurred boundaries between senses, we posit that each dictionary must make its own distinctions about to what degree it is a “splitter” or “lumper” of senses (Walter, 2010). As dictionaries have many different purposes from tiny pocket dictionaries to comprehensive multi-volume editions, it is clear that not every dictionary will place itself at the same point in the spectrum. As such, we view the distinction as being a case of parameters in the process of sense distinction and we claim it is necessary to find a set of parameters that can be supported by quantitative and qualitative evidence from one of our four methods, i.e., formal, cognitive, distributional, and cross-cultural. Examples of some of the parameters are:

- Does this dictionary model the systematic polysemy between an animal and its meat?
- Does this dictionary consider a transitive and intransitive meaning of a verb such as “eat” to be distinct?
- What collocations or word associations distinguish two meanings of a word?
- The degree of underspecification (McShane et al., 2005) of senses in the dictionary.
- Minimal number of attestations of a sense to be included.

**Figure 2:**
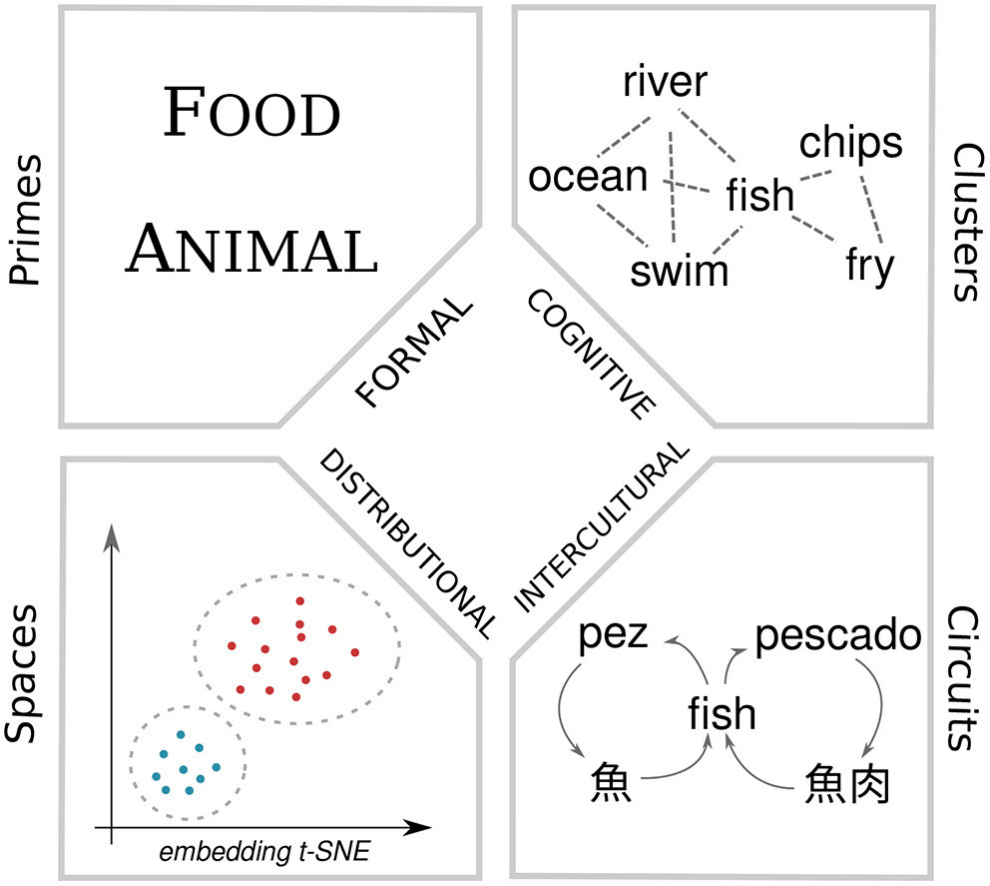
Methodologies for making sense distinctions.

In Figure 2, we define four primary elements that distinguish senses according to these four perspectives. For formal models, semantic primes (Goddard and Wierzbicka, 2013), the minimal defined elements of the representation distinguish meanings of a word. For cognitive methods, senses are defined by clusters of closely associated words in the word association graph. The distributional approaches we make are based on contextual word embeddings and these can be clustered into senses and hence divided into conceptual spaces (Gärdenfors, 2004). Finally, intercultural senses are created by circuits of translations in the translation graph. A fundamental open question is if these four different elements make the same sense distinctions for a word and how they could be integrated into a single method for making sense distinctions.

### 3.1. Formal Polysemy

Formal polysemy is perhaps the most obvious and widely used method for making sense distinctions in practice as it relies principally on human reasoning^3^. In this case, distinctions are made based on the definitions of concepts, for example definitions of “wing” such as “a movable organ for flying” and “a stage area out of sight of the audience”^4^ are obviously different to any lexicographer and would be widely recognized as distinct senses to any speaker of English. These distinctions have mostly been formalized through the use of ontologies with some success (Curtis et al., 2006; Prokofyev et al., 2013). In this case, this method is highly successful as we can see that the first sense refers to a physical object and the second to a location and these are distinct elements in ontologies such as DOLCE (Gangemi et al., 2002), where the former would be a *Non-agentive physical object (NAPO)* and the latter a *Place (PL)*. This distinction in the top-level hierarchy of DOLCE indicates that these are two distinct senses. Similarly, SUMO (Niles and Pease, 2003)^5^ is an ontology of top-level categories, however it provides a much wider coverage of the language as it consists of 20,000 terms and mappings to 117,000 WordNet senses, in place of DOLCE's 100 terms. In SUMO, these two senses of wing are also placed differently in the hierarchy with the two immediate superclasses being “limb” and “room,” both of which ultimately are subclasses of the general idea of “object.”

**Figure 3:**
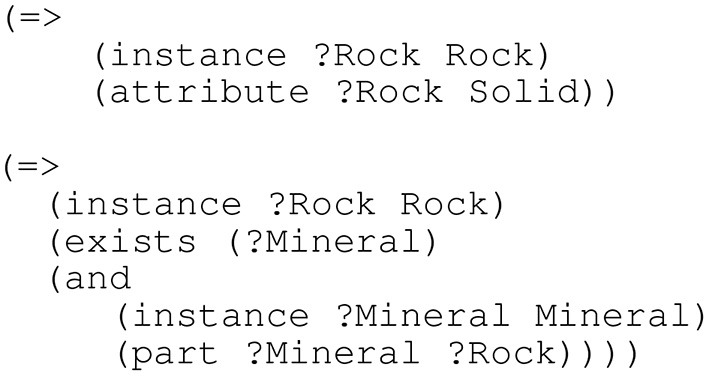
SUMO's modeling of “rock”.

That being said, relying solely on hierarchical distinctions in order to make senses is not sufficient alone; firstly because it may be the case that senses that a lexicographer would like to distinguish, which do not have a taxonomic distinction, such as “wing” meaning “one of the horizontal airfoils on either side of the fuselage of an airplane”, would also be an NAPO in the DOLCE ontology. In order to solve this, lexicographers are recommended to use definitions that consist of not only a *genus* (the class of something, such as a “sheep” is an animal) but also at least one *differentia* (a unique characteristic of the concept, such as having wool) (Hartmann and James, 1998, p. 44). However, there is no clear idea about what particular differentia would constitute a meaningful sense distinction so it is hard to decide when to make a sense distinction. The second problem is more significant in that there are many cases where large differences in the genus of a term might not naturally constitute a secondary sense. For example, “rock” can refer to a single piece of rock, which would be a NAPO in the DOLCE taxonomy, but also to a material which would be *amount of matter (M)* in the DOLCE taxonomy^6^. This is an instance of systematic polysemy where we coerce a reference of a material to an object made of that material, for example, if I say “bring me the *M*,” where *M* is a material, you understand that as the object made of that material. As such, this distinction could be considered unnecessary yet most dictionaries happily make this distinction for the word “rock,” but they are much less likely to do so for more specific words such as “crystal.” For example, Merriam-Webster, Oxford and Wiktionary, make this distinction for “rock” but not “crystal,” with English WordNet being one of the few that do for both words. SUMO has classes (“substance” and a “corpuscular object”) that allow this distinction to be stated explicitly, although the current mapping maps both WordNet senses of “rock” to the same concept which is subsumed by the class “substance,” leading to the distinction being implicit in the mappings. This is based on the assumption that named concepts in SUMO are analogous to dictionary senses, as SUMO is a formal model and treating it as a dictionary misses many of SUMO's features. SUMO as a formal ontology does not need to make this distinction explicitly as it can be inferred by its associated NLP system, SigmaNLP, in a process analogous to systematic polysemy discussed below. That is, if a reader reads a dictionary entry that does not make a substance-object distinction she is capable of inferring the implicit sense and as such the human intelligence process of the generative lexicon is analogous to the artificial intelligence method of SigmaNLP. Similarly, many dictionaries do not make the substance-object distinction explicitly and SUMO does not make it explicitly either for this reason but provides formal definitions such as in Figure 3, that provide definitions of concepts, in this case, that a rock is a solid composed of one or more minerals.

At this point, it is important to introduce the school of thought that views different senses to be related in some cases and therefore to be derivable from each other in a predictable or systematic way. Consider for instance the related senses of “chicken” (animal) and “chicken” (meat), a derivational process which can, in principle, be replicated for any animal. That is, given a noun that denotes an animal (has the sense “animal”), we can predict in a systematic way that this noun can also denote the meat of this animal (has the sense “meat”). We can identify a similar pattern for plants and their fruits (cherry tree and cherry fruit), plants or animals and the material that can be made from them (cotton plant and cotton material; crocodile animal and crocodile material) and many others, see Apresjan (1974) or CORELEX (Buitelaar, 1998) for other examples.

Given that this process can be viewed as systematic, researchers in lexical semantics that studied this phenomenon have phrased this as “systematic polysemy” (Nunberg, 1992) or also as “regular polysemy” (Apresjan, 1974) or “logical polysemy” (Pustejovsky and Bouillon, 1995). In the case of Pustejovsky, the “logical” nature of this form of polysemy originates out of the predictable manner in which different senses can be generated from an underlying abstract meaning representation, known as “qualia structure” (Pustejovsky, 1998). Qualia structure represents the meaning of a noun by way of four “qualia roles,” which are Formal, Constitutive, Telic, and Agentive, each of which representing a core semantic aspect of any noun. According to Pustejovsky's Generative Lexicon theory, word senses can be dynamically generated from this representation on the basis of compositional semantic requirements. In this context, the notion of “type coercion,” introduced by Pustejovsky to explain how words can acquire a different sense (i.e., “change semantic type”) if the compositional semantic structure to which it contributes requires this, is also relevant.

In order to fully expand a formal model of senses, we need to consider both the genus through theories such as DOLCE but also the differentia in a way that the different qualities of a sense are taken into account. An approach for this may be through a formal language such as Abstract Meaning Representation (Banarescu et al., 2013, AMR), which has been shown to be a robust method for the representation of syntax. It is possible that using robust approaches for parsing (Blloshmi et al., 2020) could be applied to existing dictionary definitions to allow for more formal reasoning. However, there remain two major issues to the more formal analysis of meaning by a language such as AMR. Firstly, there are many equivalent phrasings of the same definition, for example “father-in-law” could be defined by several definitions including:

- father of spouse
- male parent of spouse
- father of wife or husband
- male parent of wife or husband

Secondly, as we will show below with the analysis of “fish,” the actual set of differentiae can vary in importance and it is hard to check which differentiae are essential to the sense.

### 3.2. Cognitive Polysemy

As sense distinctions are fundamentally a function of cognitive action, it makes sense to look for evidence of sense distinctions from cognitive experiments. The most direct way to do this with current technology is through direct measurement of brain activity using methods such as Functional Magnetic Resonance Imaging (fMRI) to directly see if there are distinctions between different senses of words. Copland et al. (2007) was a study that did exactly this by looking at differences in brain activation between two senses of “bank” using priming concepts such as “money” and “river” concluding that there are clear differences between these two senses. This supports the hypothesis that there are cognitive differences in how we approach homonyms. However, it is less clear if the more subtle sense distinctions that lexicographers make can be clearly distinguished with such technologies, and similar studies have difficulty in detecting similar distinctions due to the fact that semantically related words “recruit similar regions” of the brain (Sachs et al., 2011). Other approaches such as priming or directly asking participants have also been investigated in the context of semantic ambiguity (Hino et al., 2006), but these have not yet been successfully applied to the task of making sense distinctions. Of course, directly asking participants if they think words have the same meaning would directly find sense distinctions but is unlikely to be financially viable for all senses in a modern lexicographic workflow.

Given the challenges with such research, much work has attempted to understand cognitive connections between concepts in the brain by means of word association games, which are an effective and cheap way to measure cognitive associations (Szalay and Deese, 1978). Recently a large database of such associations has been introduced called the “Small World of Words” (De Deyne et al., 2019, SWOW), which allows us to directly study the associations made by thousands of speakers of English and 14 other languages. The natural method of making this analysis is to look for clusters within the graph by means of algorithms for *community detection* (Fortunato, 2010), which detects highly connected subgraphs. It is natural to suppose that these clusters would correspond to senses within the graph, for example an ambiguous word like “bank” is connected to many other words that are closely related to each other as well such as “money,” “account,” “teller,” and “save.” Meanwhile there are other connections listed in SWOW that do not have any other further connections to this cluster such as “river” and “water” and smaller senses such as “piggy” and “sperm.” As such, it seems that such an analysis will naturally lead to the detection of homonyms but it is less clear that more subtle sense distinctions can be inferred.

A recent study by Branco et al. (2020) has shown that graph-based analysis using the SWOW word association norm database can outperform even state-of-the-art word embedding models at predicting word similarity and provide competitive performance on tasks such as natural language inference with state-of-the-art methods. Although it is clear that the database does effectively capture sense distinctions that are widely used, there are also reasons to be sceptical about the information in this database for the task of making sense distinctions.

Firstly, it is clear that the word association database consists of a large degree of collocations and this introduces a bias in the database, for example “bank” is the most used term when primed with the word “piggy” but the converse is much less frequent, that is “piggy” is rarely suggested for the prime “bank.” Secondly, there are word senses such as “bank” meaning a “flight manoeuvre” that have no clear relation to any of the word associations. Further, it seems possible that some sense distinctions may be difficult to capture with word associations as they refer to unlexicalized concepts such as certain kinds of movements. We also note that these databases normally don't distinguish different parts of speech in their data, so it is necessary to disaggregate the senses by part of speech as well. So, it may be the case that certain sense distinctions cannot be detected with this cognitive approach. Branco et al. (2020)'s study calls for “a unified account of lexical semantics” and it seems that there are certainly strong synergies between the cognitive approach and the distributional method described in the next section, as both are able to effectively detect collocations. In fact, there has already been some work in automatically inferring word association norms (Reyes-Magaña et al., 2020) based on distributional word embedding models and the success of this suggests that the information captured by the models is very similar.

Another approach that holds some promise is the mapping of the senses directly with areas of the brain such as in the work of Kocoń and Maziarz (2021), where the areas of the brain are directly connected with the semantic graph of WordNet. The addition of such connections allows for a graph representation that performs better at NLP tasks than just the semantic network alone. As such, it seems that direct mapping of semantic senses with cognitive regions can be helpful in building semantic networks and thus making sense distinctions.

### 3.3. Distributional Polysemy

The distributional hypothesis that “you shall know a word by the company it keeps” (Firth, 1957) has quickly become the dominant paradigm within computational linguistics and natural language processing. In particular, this has been due to the emergence of word embedding model such as word2vec (Mikolov et al., 2018). These models rely on the distributional context of a word and convert them to a vector form that is readily usable for a wide range of further applications. In this way, these models can be considered as more advanced versions of the collocation-based methods that are commonly used to make sense distinctions and offer more discriminative power at the cost of leading to results that are difficult to interpret and explain. The first word embedding models simply generated a single vector for each word, ignoring heteronyms, part-of-speech, and other distinctions that a lexicographer would typically make. However, it was quickly seen that such models were limited by not identifying senses and attempts were made to produce distinct vectors for each sense based on existing sense catalogues such as WordNet, e.g., the AutoExtend method of Rothe and Schütze (2015). More recently, contextual word embeddings models, most notably the BERT model (Devlin et al., 2019), have become popular and these models create a distinct vector for each occurrence of a word. Recent studies have shown that these vectors are easily clustered into broad sense distinctions such as homonyms (Nair et al., 2020), but they have also shown that finer-grained sense distinctions are much less obvious from these works.

**Figure 4:**
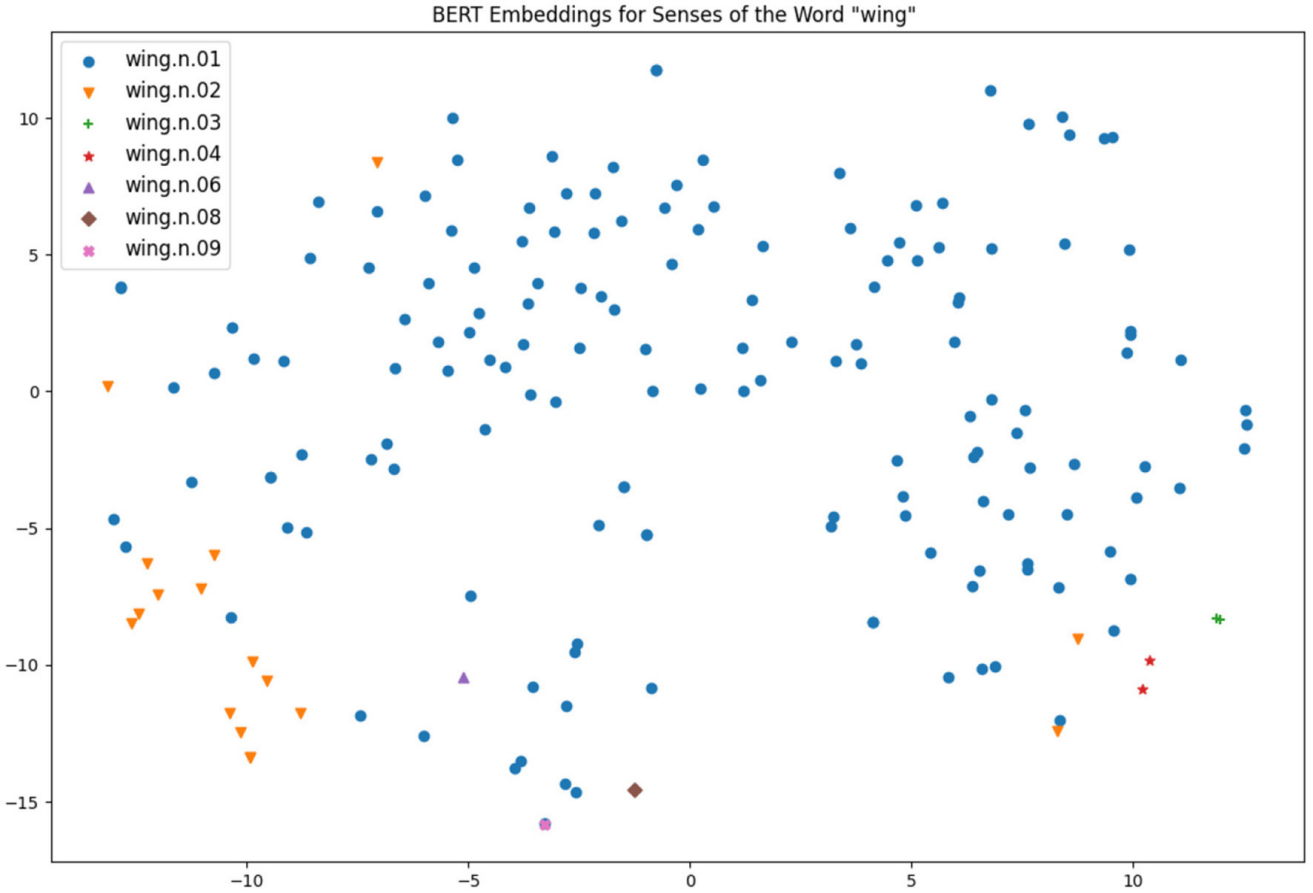
Visualizations of BERT embeddings for different uses of wings.

**Figure 5:**
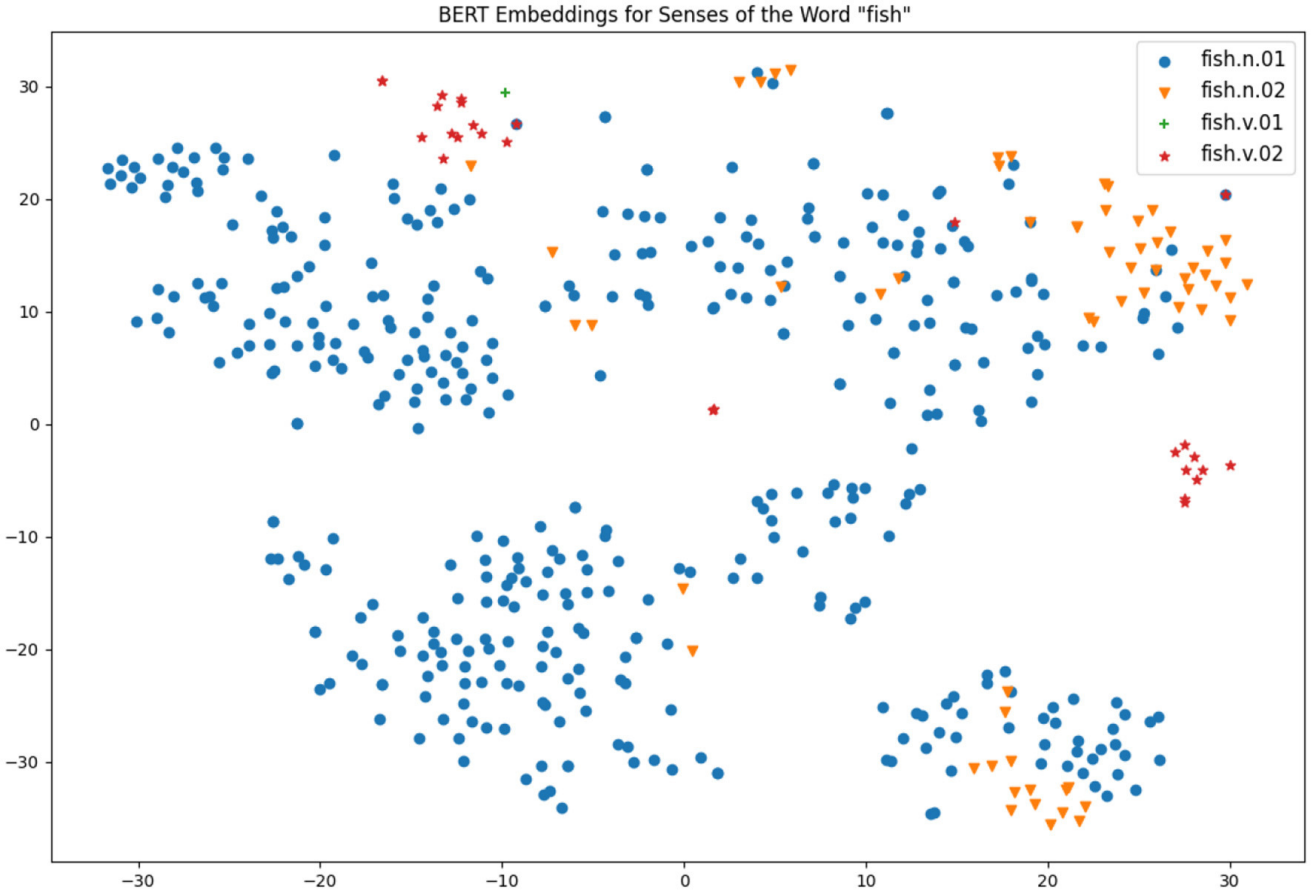
Visualizations of BERT embeddings for different uses of fish.

Two particularly interesting works from the same research group have also shed light on the connection of contextual word embeddings and word senses: firstly, Scarlini et al. (2020) showed that the usage of an existing sense catalogue such as BabelNet (Navigli and Ponzetto, 2012), which is based on WordNet and Wikipedia as principal sources, can improve the quality of the sense embeddings created. Secondly, Generationary (Bevilacqua et al., 2020) is another system that could infer natural language definitions from contextual word embeddings and it was shown that the definitions were effectively very similar to the definitions given in a traditional dictionary. As such, it seems clear that there is much information captured by these models and they can be an effective method for defining sense distinctions, but due to the obtuse nature of these vectors it can be hard to explain the results of such systems. A common approach is to reduce these highly multidimensional vectors to a 2-dimensional space so they can be visualized easily, by means of a method such as *t-Distributed Stochastic Neighbor Embedding (t-SNE)* (Van der Maaten and Hinton, 2008). That being said, such a representation is too simplistic to make good sense distinctions, as we will see below in Figures 4, 5.

While there may be potential for distributional methods to be a nearly universal solution to making sense distinctions, there are still some weaknesses of the method. Firstly, they often struggle with less frequent senses, especially if this less frequent sense is not primarily used in specific collocations. Secondly, the interaction of systematic polysemy and distributional models is not clear. For example, systematic polysemy such as the food-animal distinction is clear in a distributional model as for example “fish” may co-occur with words like “chips” or “swim” and these can easily be separated to deduce these senses, yet for organization-building distinctions such as for “school,” it is less clear if there are co-occurring words that would make this distinction. Finally, these distributional models have the tendency to be *black boxes* where the results are not easy to explain and so it is challenging to see how they may be accepted by a working lexicographer along with their other tools.

### 3.4. Intercultural Polysemy

A final principle that is used to make sense distinctions is by looking at evidence from other languages in order to make sense distinctions. For example, the homonymous senses of “bank” can easily be distinguished as there are very few other languages that use the same word for both a financial bank and a river bank. Similarly, languages that do not make a food-animal distinction can use the evidence that English makes a distinction such as “mutton”/“sheep” to provide evidence for such distinctions. However, the question here is whether it really makes sense to rely on another language to make a sense distinction. For instance, there are a great number of languages that distinguish lexically between male and female role words, e.g., “teacher” must be translated with respect to the gender of the people being referred to in German, French, Spanish, Italian, and many other languages, and it does not seem that this is a difference that a speaker of language such as English would consider important.

For bilingual dictionaries, the notion of distinct senses is dependent on the nature of the translations to another language. That is, an English-German dictionary would not list the translation of “fish” to “*Fisch*” twice to account for the food-animal polysemy, while an English-Spanish dictionary would have to, as Spanish has two translations (“*pez*” and “*pescado*”) according to this distinction. In the context of monolingual dictionaries, it seems much less certain as to whether such sense distinctions are appropriate and represent real distinctions that would be made by native speakers of that language. Further, the data we have presented here is mostly on European languages and the effects of using languages from different families needs further investigation although it seems likely that sense distinctions would be clearer across very different languages.

On the other hand, translation data is abundant due to the existence of large parallel corpora used in machine translation as well as large multilingual lexical resources such as BabelNet (Navigli and Ponzetto, 2012) and Apertium (Forcada et al., 2011; Gracia et al., 2018). As such, it seems natural that the use of these resources can provide important evidence for translation and an approach by means of translation graphs and clustering algorithms could be highly effective. It should also be noted that the use of parallel texts has already been shown as an effective method for distinguishing senses and the “one homonym per translation” hypothesis (Hauer and Kondrak, 2020) closely matched the “one sense per discourse” model (Gale et al., 1992) already used as a principle for making sense distinctions.

### 3.5. Metaphors and Metonyms

We should also note the limits of methodologies for making sense distinctions as a dictionary cannot truly cover all usages of a sense that may occur in the corpus. This is due to the productive nature of language and the fact that new senses are continuously being created. It is common, especially in poetic language, to introduce metaphorical senses that are unlikely to be found in dictionaries. This is often the process by which new words are created as described by Nunberg (1987):

Metaphors begin their lives as novel poetic creations with marked rhetorical effects, whose comprehension requires a special imaginative leap. As time goes by, they become a part of general usage, their comprehension becomes more automatic, and their rhetorical effect is dulled.

As an example, the English word “overwhelm” has gradually lost its original meaning of “to flood (over),” to the point that the antonym “underwhelm” could enter common usage and many native speakers even speculate on the original meaning^7^. Such metaphors are conventionalized and can be considered as a case of non-systematic polysemy, however many other metaphors are productive and impossible to capture with a fixed list of senses in a dictionary. Metonymy is a very similar process distinguished by the fact that the new sense is mapped to a concept within the same domain (Gibbs, 1999), and is thus generally less of a conscious decision by the author than metaphor. Recent results have shown strong results in the detection (Shutova, 2015; Zayed et al., 2020a) and the interpretation of metaphors (Zayed et al., 2020b). Therefore, a system for making sense distinctions should also be aware of metaphor and metonymy and be able to explain this to the user.

### 3.6. An Integrative Approach

The issue of sense distinctions is one of primary importance for lexicographers and the idea of dictionaries as authorities in language is undermined by the wide variety of sense catalogues found in different dictionaries. The idea that this is simply due to distinctions between “splitters” and “lumpers” seems questionable as there is a lot of variance in the number of senses a dictionary has and computational users of dictionaries have been highly critical of the inventories in extant resources, especially WordNet^8^. As such, it would be highly useful for a corpus-based system that lexicographers could use to analyse the meanings of words and form them into clusters.

The current state-of-the-art in distributional semantic methods, especially with respect to contextual word embedding methods, seems like it would be able to satisfy this goal. However, a number of limitations exist, including that the black-box nature of this method is not easy to translate into a word sense catalogue. Therefore, it seems that the power of the distributional method could be used to infer a formal representation of senses, such as by combining the methodology of Generationary with more formal approaches such as Abstract Meaning Representations. These methods could be further augmented with information from cognitive databases such as Small World of Words as well as multilingual parallel texts, but there are still major practical challenges in this. Moreover, it is necessary that such a system is adaptable to the needs of a given lexicographic project and can be tuned by means of parameters that fit the goals of the particular dictionary. In the remainder of this paper, we will look at how the theories can be applied for making sense distinctions and sketch the possibilities and challenges by combining them.

## 4. Case Studies

**Table:** 

	***Fish* (noun)**	***Fish* (verb)**	***Wing* (noun)**	***Wing* (verb)**
English WordNet	2	2	11	1
Wiktionary	17 (3 homonyms)	8	28	8
Merriam-Webster	4	5	12	5
Lexico.com (OUP)	3 (2 homonyms)	4	10	3
Cambridge	2	3	6	1
Dictionary.com	9	10	24	13
Collins	2	3	9	5

As case studies, we have selected two common English nouns that exemplify the difficulties in making sense distinctions and sit in the middle of our spectrum of polysemy. The first noun, “fish”, was chosen because it is relatively clear in the meanings that are frequently used, with all dictionaries identifying the sense as an animal and as a food for the noun and the meaning of “to catch fish” as well as metaphorical extensions of this meaning such as in “fishing for compliments.” Many dictionaries also record other senses distinguishing between “fish” referring specifically to vertebrate aquatic animals and another for invertebrates such as jellyfish, shellfish, as well as many extended senses and deverbal forms, notably the meaning of the target of a scam. Wiktionary^9^ also describes three homonyms in the entry differentiating between the noun and verb senses due to a different but morphologically-related form in Old English and a third rare homonym as an Anglicization of the French “fiche” meaning “a counter used in various games,” a homonym that is also found in OUP's Lexico.com, but defined in this case as “A flat plate that is fixed on a beam or across a joint in order to give additional strength.”

We selected “wing” as a second word as there are a large number of senses although most of them can be described as variations on “the side of something,” e.g., birds/insects, planes, buildings, political organizations, football squad formations. The verb senses of “wing” have a very high degree of disagreement among dictionaries. An analysis of the first 100 verbs matches in the English TenTen corpus on Sketch Engine (Jakub́ıček et al., 2013) reveals that the sense of “equipped with wings” is the most frequent with 80 (85.1%) of the occurrences^10^, 6 (6.4%) used the sense of “flying with wings” and six occurrences (6.4%) were the British slang usage “winging it,” meaning *to succeed without due preparation*. The remaining two usages were senses likely uniquely invented by their authors, namely “West Winging it” and “in melody winged over.” All dictionaries, except for Wiktionary, miss one of the three most frequent senses although some dictionaries recognize the most frequent sense as an adjective only (“winged”), which fits with the corpus usage observed above.

### 4.1. Formal Analysis

The formal analysis of the words is based on the existing definitions given in dictionaries as there is still much to be examined about more computational approaches such as Abstract Meaning Representation. We first look at the genera of “fish” in Table 1 and we list the noun and verb senses of the word as they appear in WordNet. These are then further distinguished in a more formal ontology, in this case SUMO (Niles and Pease, 2001).

**Table 1:** WordNet senses with SUMO mappings for the noun and verb “fish.”

**POS**	**Wordnet sense**	**Direct hypernym**	**Indirect hypernyms**	**SUMO Mappings**
Noun	Any of various mostly cold-blooded aquatic vertebrates usually having scales and breathing through gills	Aquatic_vertebrate	Craniate, vertebrate < chordate < animal, animate_being, beast, brute, creature, fauna < being, organism < animate_thing, living_thing < unit, whole < object, physical_object < physical_entity < entity	Fish (equivalent mapping)
Noun	The flesh of fish used as food	Food, solid_food	Solid < matter < physical_entity < entity	FishMeat (subsuming mapping)
Verb	Catch or try to catch fish or shellfish	Catch, grab, take_hold_of	< Clutch, prehend, seize < get_hold_of, take	Fishing (equivalent mapping)
Verb	Seek indirectly	Look_for, search, seek		Investigating (subsuming mapping)

In contrast to “fish,” the word “wing” has multiple unrelated senses in WordNet, as can be seen in Table 2. Most of these are clearly differentiated by the genus, with there being seven main senses identified: organ, artefact, grouping, hockey player, meat, flight formation, and addition. Some of these categories, however, seem somewhat arbitrary; a “wing” in the sense of a flight formation (10.), while semantically related to “flank” (6.), does not share any hypernyms with the latter, apart from the highly abstract “entity.” In other words, a formal analysis, at least when considered from the viewpoint of ontology and hypernymy, does not always seem to clearly and neatly capture the semantic inter-relationship between word senses.

**Table 2:** WordNet senses with SUMO mappings for the noun “wing” categorized according to hypernyms.

**Wordnet sense**	**Direct hypernym**	**Indirect hypernyms**	**SUMO Mappings**
1. A movable organ for flying	Organ (a fully differentiated structural and functional unit in an animal that is specialized for some particular function)	Piece < thing < physical_entity < entity	Organ (subsuming mapping)
2. Wing (one of the horizontal airfoils on either side of the fuselage of an airplane)	Airfoil, aerofoil, control surface, surface (a device that provides reactive force when in motion relative to the surrounding air; can lift or control a plane in flight)	Device < instrumentality, instrumentation < **artefact, artifact** < **unit, whole** < object, physical_object < physical_entity < entity	WingDevice (subsuming mapping)
3. Wing, offstage, backstage (a stage area out of sight of the audience)	Stage (a large platform on which people can stand and can be seen by an audience)	Platform < horizontal_surface, level < surface < **artefact, artifact** < **unit, whole** < …	PerformanceStageWing (equivalent mapping)
4. Fender, wing (a barrier that surrounds the wheels of a vehicle to block splashing water or mud)	Barrier	Impediment, impedimenta, obstructer, obstruction, obstructor < construction, structure < **artefact, artifact** < **unit, whole** < …	EngineeringComponent (subsuming mapping)
5. Wing (a unit of military aircraft)	Air unit (a military unit that is part of the airforce)	Force, military_force, military_group, military_unit < social_unit, unit < organization, organization < **social_group** < **group, grouping** < **abstract_entity, abstraction** < entity	Organization (subsuming mapping)
6. Flank, wing (the side of military or naval formation)	Formation (an arrangement of people or things acting as a unit)	Arrangement < **group, grouping** < **abstract_entity, abstraction** < entity	GroupOfPeople (subsuming mapping)
7. A group within a political party or legislature or other organization that holds distinct views or has a particular function	**social group (people sharing some social relation)**	**Group, grouping** < **abstract_entity, abstraction** < entity	Group (subsuming mapping)
8. Wing (a hockey player stationed in a forward position on either side)	Hockey player, ice-hockey player (an athlete who plays hockey)	Athlete, jock < contestant < individual, mortal, person, somebody, someone, soul < being, organism (1) < Animate_thing, living_thing < unit, whole < object, physical_object < physical_entity < entity (2) < Causal_agency, causal_agent, cause < physical_entity < entity	HockeyPlayer (subsuming mapping)
9. Wing (the wing of a fowl)	Helping, portion, serving (an individual quantity of food or drink taken as part of a meal)	Small_indefinite_amount, small_indefinite_quantity < indefinite_quantity < amount, measure, quantity < abstract_entity, abstraction < entity	PoultryMeat (subsuming mapping)
10. Wing ((in flight formation) a position to the side and just to the rear of another aircraft)	Place, position	Point < location < object, physical_object < physical_entity < entity	PositionalAttribute (subsuming mapping)
11. Annex, annexe, extension, wing (an addition that extends a main building)	Addition, add-on, improver (a component that is added to something to improve it)	Component, constituent, element < part, portion < object, physical_object < physical_entity < entity	BuildingUnit (subsuming mapping)

*Non-top-level hypernyms shared across senses are displayed in bold*.

We also analyzed the differentiae, in this case for the most frequent sense of “fish” as an animal and this is summarized in Table 3. The only differentia that all the dictionaries we looked at agreed on was that fish live in water, other aspects are often missed by the definitions in one or more dictionary. As such it is clear that we cannot count on a definitive set of differentiae to distinguish between senses of words, that is the fact that three dictionaries mention scales does not indicate that the lexicographer is trying to define a distinct senses. In addition, we include the SUMO definition (Niles and Pease, 2003), where the formal axioms can be paraphrased as “a cold blooded vertebrate that inhabits water, disjoint from amphibians, and reptiles.” It is also worth noting that none of these definitions are scientifically correct as fish may be (partly) warm-blooded and not all fish have scales and a tail (Nelson et al., 2016), although of course the role of a lexicographer is to capture general usage of a language not technical distinctions. However, this emphasizes a clear challenge with a formal approach to sense distinctions, in that when we have such a wide variation in differentiae, it is difficult to infer by any automatic process which particular criteria are essential to the meaning.

**Table 3:** Analysis of listed differentiae for the sense of “fish” as an animal in different dictionaries.

	Cold-blooded^*^	**Aquatic**	**Vertebrate**	**Fins**	**Gills**	Scales^*^	Tail^*^
English WordNet	✓	✓	✓		✓	✓	
Wiktionary	✓	✓	✓	✓	✓		
Merriam-Webster	✓	✓	✓	✓	✓		
Lexico.com	✓	✓	✓	✓	✓		
Cambridge		✓			✓	✓	
Dictionary.com	✓	✓	✓	✓	✓	✓	
Collins		✓		✓			✓
SUMO	✓	✓	✓				

* *Scientifically inaccurate differentia*.

### 4.2. Cognitive Analysis

For cognitive analysis, we took the Small World of Words graph and extracted the subgraph consisting of only the words directly connected to the word we are studying. That is we took the subgraph consisting of all words that have a forward or backward association to the words “wing” or “fish,” we also discarded all terms that had <3 associations in the dataset. We then applied the Girvan-Newman community detection methodology (Girvan and Newman, 2002) to find the main clusters within the graph. We see the main clusters that have been extracted in Table 4.

**Table 4:** Girvan-Newman cluster analysis of the Small World of Words dataset.

**Clusters for “wing”**	
1	Bird, butterfly, dragonfly, feathers, flap, flutter, fly
2	Air, airplane, flew, flight, plane, propeller
3	Bat, man
4	Chicken, feather
5	Left, right
6	Angel
7	Gull
**Clusters for “fish”**	
1	Algae, amphibian, anchovy, animal, aqua, aquarium, barracuda, boat, boating, boats, brine, brook, calamari, catfish, chowder, clam, coral, crab, crayfish, creek, dive, diver, diving, dolphin, downstream, eat, eel, fighting, filter, fin, fingers, fish tank, fishy, flatfish, fleshy, flipper, flop, flounder, flying, fresh, go, goldfish, gull, harbor, harpoon, heron, hunt, Japan, jelly, kelp, lagoon, lake, lobster, Maine, mammals, marine, marines, mermaid, mollusk, mussel, ocean, octopus, oily, otter, oyster, pacific, paella, pelican, pet, pets, pie, pier, pike, poach, pond, porpoise, prawn, reef, river, sail, sailing, salamander, salmon, salty, sardine, sashimi, scales, scallop, scaly, scuba, sea, seafood, seagull, seahorse, seal, seaman, seashell, seaside, seaweed, shark, shell, shrimp, sinker, slimy, slippery, snorkel, sole, spawn, spear, spiny, squid, squish, starfish, stick, stickleback, stingray, stream, sucker, sushi, swim, swimmer, swish, sword, swordfish, taco, tadpole, tank, trout, tuna, underwater, upstream, water, whale, wharf, worm
2	Bait, bass, carp, cast, casting, catch, catchy, caught, cod, fishing, fishing pole, hook, hooked, lure, net, Norway, perch, reel, rod, tackle, tarp, troll, unhook, worms
3	Batter, chips, Friday, fried, fry, grilled, smoked
4	Bouillon, broil, dish, escargot, f, food, frying, grill, gulp, gut, market, nibble, plaice, platter, plenty, poison, protein, raw, rotten, rotting, skate, skillet, smelly, smelt, stinky
5	Angle, beta, England, Omega
6	Chip, flake, flaky, scale
7	Dill, herring, Sweden, Swedish
8	Choral, mainstream, school
9	Backbone, guts, rumble

For “wing,” we see major clusters corresponding to some of the main homonyms of the word with Cluster 1 referring to the part of an animal, Cluster 2 as a part of a plane and Cluster 5 as a political orientation. Cluster 4 is suggestive of the food sense with the association with “chicken,” although “feather” is probably not associated with this sense. The third cluster is probably erroneous due to the strong association between “bat” and “man”; however this does detect the sense of a “wing man.” It is not clear why “angel” wings are so distinct from other animal wings^11^ and Cluster 7 is identifying wing as a shape, as in “gull-wing doors.” As such, we can see that the cognitive analysis has identified six main senses of “wing,” but other smaller senses are not being detected. Other senses such as in “to wing it” are probably not being connected as the association between “wing” and “it” had only two instances in SWOW so was filtered out. There were several other highly useful associations at this level, such as “Buffalo” and “food” to support the food sense and “commander” to support the “wing man” sense; however there were many other noisy relations, such as “little” (perhaps due to the Jimi Hendrix song) and “prayer” (from the idiom “wing and a prayer”).

The analysis of “fish” provides many more associates and it seems there is more information for this word. We see at least the three main homonyms of “fish.” with the first cluster referring to the animal sense, the second cluster to the activity of catching fish and the third and fourth to the sense of foods. It is interesting to note that this analysis hints at there being a strong cognitive distinction made between “fish and chips” as a specific dish and “fish” as a more general meat, it would certainly be interesting to see if this is also seen in other languages. The remaining clusters are less clear and may in part be to do with errors made by the annotators of SWOW, e.g., the strong association with “choral” is almost certainly due to annotators misreading it as “coral.” It should also be noted that some of the clusters have a very weak association between the elements such as Cluster 5, and more investigation of the algorithm would help in detecting senses here. Similarly, we note that Cluster 7 is created for the sense of “Swedish Fish,” a candy in the US, but this cluster then pulls in other Sweden-related words from other senses. It is also interesting to note how certain words support different senses, for example we see that Japan and Maine are associated with the animal sense, whereas Norway is associated with the fishing sense. Similarly, some species of fish are thought of more as animals, some as food (e.g., “plaice,” “skate”) and some as for recreational fishing (e.g., “carp,” “bass”).

### 4.3. Distributional Analysis

For distributional analysis, we use the BERT model as the basis of the analysis. This model was selected as it has been shown to have strong performance across a wide number of tasks for senses (Bevilacqua et al., 2020; Nair et al., 2020), however given the rapid development of language models, it is possible that results may improve rapidly over the next few years as stronger models are developed. We took the Gloss Tag corpus that is released as part of Princeton WordNet (Fellbaum, 2010) as this corpus has annotated each word with the sense in WordNet allowing us to analyse each individual occurrence. Note that this corpus consists of annotated definitions from Princeton WordNet; however we just treat each definition as a free-standing sentence. The senses, their definitions in Princeton WordNet and frequency in the corpus are given in Table 5. While these definitions may not fully reflect standard language usage, it was chosen as it is sufficiently large and annotated to a very high quality with a focus on less frequent senses. We also note that “very few large annotated datasets [for WSD] are available” (Taghipour and Ng, 2015) one and semi-automatically constructed datasets would be risky for this detailed analysis. For each sentence of the corpus using either the word “fish” or “wing” we applied the BERT model taking the sum of the last four hidden layers as the embedding and we extracted the vector associated with the target word. We then applied a t-SNE projection to these vectors and these are shown in Figures 4, 5.

**Table 5:** Senses appearing in the Princeton WordNet Gloss Tag corpus.

**Sense**	**Definition**	**Frequency**
Wing.n.01	A movable organ for flying (one of a pair)	156
Wing.n.02	One of the horizontal airfoils on either side of the fuselage of an airplane	17
Wing.n.03	A stage area out of sight of the audience	2
Wing.n.04	A unit of military aircraft	2
Wing.n.08	A group within a political party or legislature or other organization that holds distinct views or has a particular function	1
Wing.n.09	The wing of a fowl	1
fish.n.01	Any of various mostly cold-blooded aquatic vertebrates usually having scales and breathing through gills	414
Fish.n.02	The flesh of fish used as food	62
Fish.v.01	Seek indirectly	1
Fish.v.02	Catch or try to catch fish or shellfish	27

For the word “wing”, we see that the noun senses that are most common are clustered in the bottom right-hand corner of Figure 4, although there are a few outliers, notably the sentence “an artificial fly that has wings extending back beyond the crook of the fishhook” appears near the top of the graph and may indicate a distinct sense as this is not related to aviation. Minor senses are mostly on the bottom left-hand corner of the diagram; however there is not really enough information to make an informed decision.

Figure 5 is much more complex due to the fact that there is more data available for this. As in the previous plot, the most frequence sense appears in all parts of the t-SNE plot, but here is more clearly clustered. Part of this reason is to do with the different forms occurring in text, as the lemma “fish” appears as “fish,” “fishes,” “fishing,” and “fished” in the corpus. The large cluster on the bottom left-hand side corresponds to the form “fishes” and the small cluster of verb forms on the far right-hand side corresponds to the form “fishing.” For the second sense of fish we see most of the senses clustered in the top right-hand corner, suggesting that these senses are mostly being found correctly. However, there is a large cluster containing both “fish” as animal and “fish” as meat senses in the bottom corner. An analysis of the definitions suggests that these examples concern the catching of fish, for example, we have sentences such as “someone whose occupation is catching fish” (fish as animal) and “a small house where smoke is used to cure meat or fish” (fish as meat). As such, we see that the distributional method is focusing more on the context of the word than the formal genus of the word.

It is also important to note that although the clusters are apparent with the annotation, for the most part they would not be obvious without the sense labels and clusters often contain examples of multiple senses. As such, it is not so clear how useful such an unsupervised approach would be to lexicographers and this explains why automatic word sense induction, while a useful tool, cannot solely solve the issue of making word sense distinctions.

### 4.4. Intercultural Analysis

**Figure 6:**
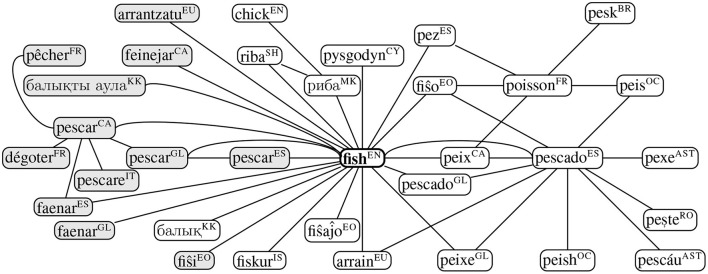
Paths starting from fish in the Apertium translation data (Goel et al., 2021) where a non-exhaustive list of translations can be retrieved by traversing individual paths. ISO 639-1 language codes are provided in superscript and verb lemmas are specified in gray.

For the intercultural analysis we take the Apertium graph of translations (Gracia et al., 2018) as the basis of our analysis. We plot the immediate neighborhoods of the word “fish” based on the translations given in this resource in Figure 6. The Apertium graph is quite incomplete and for many languages there are no translations, yet we are able to see several large cycles that correspond to some of the senses that we would expect. Firstly, we have a cycle of “*fiŝo*” (Esperanto) → “*poisson*” (French) → “*peis*” (Occitan) → “*pescado*” (Spanish) → “*peix*” (Catalan)/“*pescado*” (Galician), which corresponds to the food sense and an overlapping cycle of “*pez*” (Spanish) → “*poisson*” (French) → “*fiŝo*” (Esperanto)/“*peix*” (Catalan), which corresponds to the animal sense. We also see another cycle corresponding to the act of catching fish created by the cycle of “*pescar*” (Spanish) → “*pescar*” (Galician) → “*pescar*” (Catalan) → “*faenar*” (Spanish). This shows that it is possible to detect senses using intercultural evidence; however a more complete database of interlingual correspondences, perhaps automatically constructed from a parallel corpus, would be essential to provide useful and clear methodologies for making sense distinctions.

**Figure 7:**
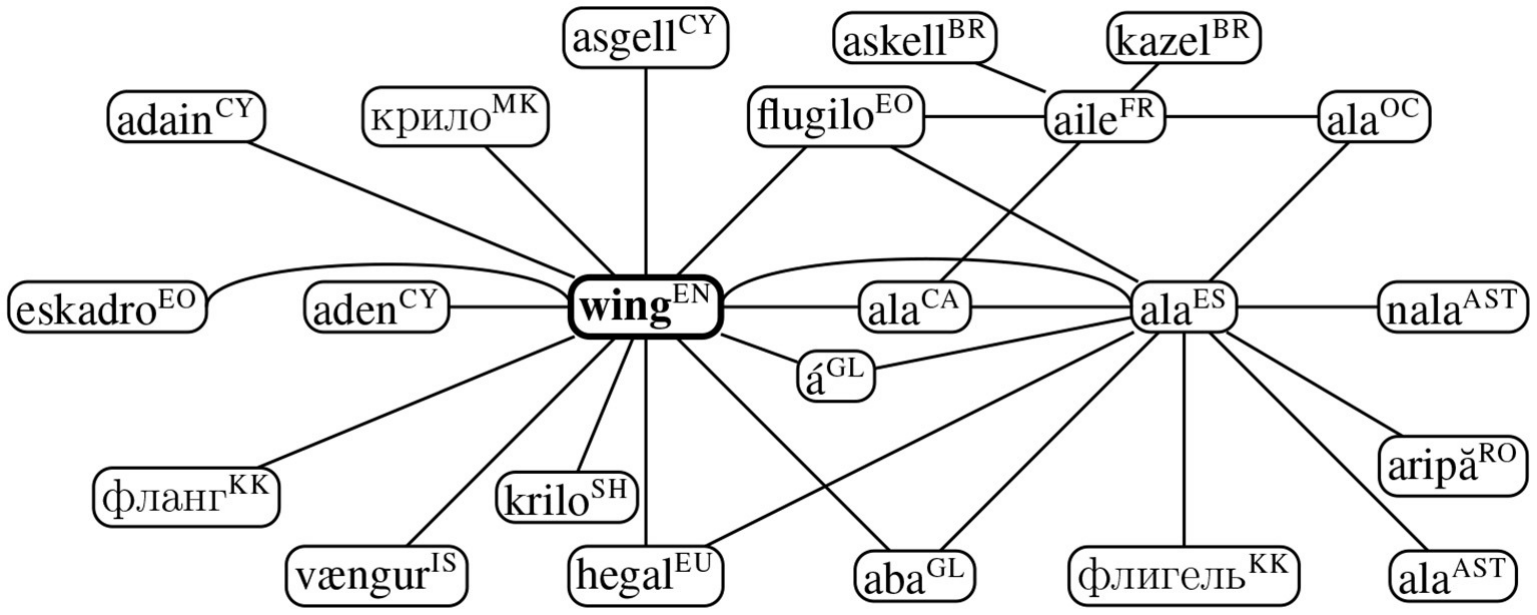
Paths starting from wing (noun) in the Apertium translation data (Goel et al., 2021) where a non-exhaustive list of translations can be retrieved by traversing individual paths. ISO 639-1 language codes are provided in superscript.

In the same vein, we construct the graph of translations of the word “wing” (noun) in Apertium dictionaries as depicted in Figure 7. Although many of the translations are associated to wing as a means of flights, as in “*ala*,” “*aile*,” and “á” in Catalan, French, and Galician, respectively, there are other connotations which are translated as different senses, such as “*eskadro*” in Esperanto which refers to a squadron. Similarly, “*kazel*” in Breton would also refer to wing as an extension of a building. In the current version of the data, we could not retrieve any translations for “wing” as a verb.

### 4.5. Unified Analysis

For the word “fish” we see that all methods are able to distinguish the animal meaning, the meat meaning and the action of fishing, suggesting that these three senses are widely used and clearly distinguished senses. The metaphorical verbal sense (as in “fishing for compliments”) is only seen formally, but this is probably due to its low frequency. For distributional methods and intercultural methods it may be possible to find this distinction with more data, but it is less clear how a cognitive method could be further extended, that is, it is not certain that distinguishing word associations such as “compliments” would start to appear when asking more users. The methods also suggest some other distinctions that are not natural from the formal approach, such as fish as a specific English dish (in the cognitive analysis) and fish as something to be hunted (in the distributional analysis). Also, the analysis of differentiae shows the limits of formal methods as it is difficult to arrive at a definition that can be widely agreed on and easily formalized such as we saw in the example of “fish,” where the dictionary definitions have significant differences from each other, SUMO and the ichthyological definition. While dictionaries are made for different purposes and in this case, it could be possible that all dictionaries should have adopted the scientific definition, it is difficult to see how this could be generalized to other senses when there is more subtle distinctions in senses created by phenomena such as systematic polysemy.

For “wing,” we have even less agreement between the models, with the distinction between an animal's wing and a plane's wing being the only solid distinction, and this does not appear in the intercultural analysis. We did a further check on multilingual resources which has not found any language that makes the distinction lexically. Meanwhile, the formal analysis shows a large number of senses with clear distinctions but this is not supported by the corpus-based analysis. As such, it can be said that “wing” is a word that can be easily coerced into new senses and requires a more nuanced formal theory such as that of the generative lexicon.

Overall, formal approaches allow for many sense distinctions to be made but when not backed by corpus evidence, this leads to senses that are unnatural to users of the dictionary^12^, especially if many of these senses are rare metaphors or semantic shifts. Cognitive approaches seem to be quite well-adapted to making sense distinctions, but there are questions about how this may scale to more infrequent sense distinctions. Distributional methods hold much promise, but the poor results and the black-box nature of these approaches raise questions about how useful it can be for a lexicographer. Finally, inter-cultural methods showed some effectiveness, but the Apertium resource here is too small and incomplete and new, large translation graphs would be necessary to fully validate this method. Further, some obvious sense distinctions are simply nonexistent in this analysis, while other sense distinctions appear that would not be obvious to a native speaker of the language in question.

## 5. Future Perspectives

Lexicography is a field that is undergoing dramatic change due to the emergence and adoption of electronic lexicography (eLexicography) and yet there are still not solutions to one of the greatest debates in lexicography, namely that of whether to “split” or “lump” senses. A fine-grained model for why lexicographers make certain distinctions and would redefine this debate among researchers and would be influential in the creation of new dictionaries. This would allow existing lexicographic projects to improve the quality of their entries and also to continue to develop new senses for words as well as defining neologisms. Further, new electronic dictionaries need sound principles on which to make sense distinctions and data-driven methods can reduce the costs of developing dictionaries enduring that resources are available for minority languages. The ELEXIS infrastructure (Krek et al., 2018, 2019), which is developing a new infrastructure for lexicography through tools such as Lexonomy (Měchura, 2017) and Sketch Engine (Kilgarriff et al., 2014), is key in leading this. As such, a better understanding of how quantitative and qualitative analysis of sense distinction is vital for understanding how we build better tools for lexicographers.

With respects to natural language processing, Princeton WordNet remains the most cited and widely-used dictionaries especially for work related to word senses in English and has enabled a lot of exciting new applications especially within natural language processing and artificial intelligence. The sense inventory of WordNet has been criticized for being at times too fine-grained, containing many duplicate or hard to distinguish senses, and quantitative evidence such as that shown above can help to justify sense distinctions. The importance of cross-lingual analysis here is particularly underlined through projects such as the Collaborative Interlingual Index (Bond et al., 2016, CILI), which aims to make a single interlingual index of all senses. There are some important questions of whether such an index exists and the comparison of intercultural sense evidence with the other approaches is of vital importance to reinforce the foundations of such approaches.

In a wider picture, the unification of different views of lexical semantics is highly interesting and would lead to new results from tackling lexical semantics from these different viewpoints. This point was also made by Branco who has called for a “unified account of lexical semantics” (Branco et al., 2020) by providing comparison and inference across different lexical semantic theories. For formal lexical semantics, there have been few large-scale verification and implementation of theories of word meaning, in particular the theory of the Generative Lexicon (GL). SUMO is one of the few examples of a formal representation of the definition of words and there is still potential that has not yet been fully explored to use this as part of the dictionary creation process. Further, it is vital to discover overlaps between GL and other theories such as NSM and Semantic Web standards such as OWL (McGuinness and Van Harmelen, 2004). For cognitive lexical semantics, further validation of the models is required in particular for models of fine-grained semantics, while in contrast most studies have only looked at homonymy (Cuyckens and Zawada, 2001; Copland et al., 2007).

Distributional semantics is an area of linguistics that is currently seeing a lot of research and is evolving very rapidly, thus may offer the solution to many of the challenges of sense distinction, but there are also limitations of the theories. With the growing power of AI methods based on distributional semantics, there are some who believe that distributional models may be sufficient to solve all problems in semantics (Wang et al., 2019), yet it has been shown that the incorporation of formal models (Loureiro and Jorge, 2019), or cognitive models (Branco et al., 2020) improves model performance. It is necessary to analyse exactly what distributional models cannot do and develop new models that cover the weaknesses of straightforward distributional models. It is possible that models of *word sense induction* could fully automate the process of finding senses; however these models are often hard to interpret (Panchenko et al., 2017) and fail for fine-grained distinctions (Nair et al., 2020). Creating a bridge between linguistics and computer science by developing interdisciplinary theories that bring the results of state-of-the-art NLP models to linguistic theories will provide empirical validation or counter-examples and thus justifiable explanations for sense distinction made by automatic systems.

Finally, cross-cultural models of semantics is an area that is not sufficiently studied yet, but is likely of huge significance for answering some key anthropological questions. In particular, we are still not sure about how meaning transfers between different languages and cultures (Gracia et al., 2012). In particular, questions about the typology of languages, which is currently mostly limited to more easily observable syntactic, phonetic and orthographic features, could be deepened by fine-grained intercultural semantic modeling to provide a new range of typological features for further research. Secondly, these features would also be essential for studying language change in terms of semantics and new models for representing diachronic dictionaries that can be adopted to enable more quantitative research in historical linguistics.

The NLP task that is of particular relevance here is word sense disambiguation, where there are fundamental limits due to the difference in sense granularities, which has been a persistent issue for the task (Navigli, 2006; Dandala et al., 2013). New inventories would fundamentally change this task so that performance can be measured simultaneously at multiple levels of granularity over those methods based on the existing WordNet catalogue. More widely, WordNet is used as a tool for a wide variety of NLP tasks, so improvements in the quality of WordNet will directly improve NLP task performance. A specific example of this is sense linking (Ahmadi et al., 2020; Ahmadi and McCrae, 2021), which is a challenging mixture of semantic similarity and logical inference and better understanding of how to link senses by understanding what senses fundamentally are.

There is a need in cognitive sciences to develop theories that shed light on how humans conceptualize senses and the interaction with cognitive evidence will make theories from formal, distributional, and cross-cultural methodologies compatible with existing cognitive theories. A particular example of this is the area of language acquisition first in terms of child language acquisition, where cognitive evidence could be connected with how language is learned. Understanding sense distinction will lead to new theories of second language acquisition, by means of the interplay of the cross-cultural and cognitive models, which will lead to a better understanding of how semantics transfer across languages and how this can help learners of a second language.

We could also see research on sense distinctions as having wider impact, including commercial impact in terms of supporting existing commercial lexicography. Further, it is our experience that issues related to harmonizing meaning and sense are a common issue in large enterprises, as we described recently (Pereira et al., 2019). In particular, the development of enterprize knowledge graphs (Gómez-Pérez et al., 2017) is of increasing importance in large enterprises and the techniques of working with them including inferring senses and linking senses require understanding how to make fine sense distinctions.

## 6. Conclusion

In this paper, we have examined the task of finding an inventory of senses for a particular word through four different semantic approaches: formal, cognitive, distributional, and intercultural. Formal methods seem essential as they connect with the written definitions given in dictionaries that are the basis for how dictionaries are used. Formal methods still have many challenges including the difficulty of inferring equivalence between two definitions and we hope that by formal analysis using languages such as Abstract Meaning Representation and the distinction of *genus* and *differentiae*, it is possible to find sound sense inventories. We also note that formal approaches tend to develop larger sense inventories, in part due to the generative nature of the lexicon, but also as many dictionaries list plausible senses that are not supported by corpus evidence. Cognitive approaches, in contrast, seem quite capable of detecting the major senses of a word and provide strong evidence of the importance of sense distinctions that may not be obvious in the formal analysis, for example our results suggest that the “fish” of “fish and chips” is important enough to distinguish as a sense distinct from fish meat in other dishes. However, these approaches also seem to be limited in terms of collecting less frequent senses, such as “bank” as a flight manoeuvre and our results suggest that they may be prone to picking up idioms and other cognitive associations.

Moreover, distributional methods are among the most promising due to the rise of methods such as BERT in natural language processing. There has also been the most research in connecting them with formal theories (Bevilacqua et al., 2020) and cognitive theories (Reyes-Magaña et al., 2020) suggesting the start of a single theory of senses that can account for evidence from multiple sources. However, distributional methods struggle with infrequent or subtle sense distinctions to a greater extent than cognitive approaches. Finally, intercultural analysis can be considered as an extension of distributional analysis to a multilingual context and this offers much promise but is as of yet still not well-researched. While all these methods have challenges there is still a large potential for these methods to be used individually by lexicographers, however a single combined theory of sense distinctions that can provide a practical methodology for working lexicographers would help significantly not only in lexicography but also in a wider context across computational, cognitive and linguistic domains and this paper aims to show how we may arrive at such a theory.

## Data Availability Statement

Publicly available datasets were analyzed in this study. This data can be found at: https://smallworldofwords.org/en/project; http://linguistic.linkeddata.es/apertium/; http://en-word.net/; https://wordnet.princeton.edu/.

## Author Contributions

All authors engaged in the writing and conceptualization of the article. JM led the writing of the article and is responsible for most of the text. TF carried out the analysis for section 4.1, KG for section 4.3, and SA for section 4.4. All authors contributed to the article and approved the submitted version.

## Funding

This work was funded in part by Enterprise Ireland under Grant Number CS-2020-2119, the Irish Research Council under grant number IRCLA/2017/129 (CARDAMOM-Comparative Deep Models of Language for Minority and Historical Languages) and the EU's Horizon 2020 Research and Innovation programme through the ELEXIS project under grant agreement No. 731015. It is co-funded by Science Foundation Ireland (SFI) under Grant Number SFI/12/RC/2289_P2 (Insight_2).

## Conflict of Interest

The authors declare that the research was conducted in the absence of any commercial or financial relationships that could be construed as a potential conflict of interest.

## Publisher's Note

All claims expressed in this article are solely those of the authors and do not necessarily represent those of their affiliated organizations, or those of the publisher, the editors and the reviewers. Any product that may be evaluated in this article, or claim that may be made by its manufacturer, is not guaranteed or endorsed by the publisher.

## Footnotes

^1^ https://smallworldofwords.org

^2^We include synecdoches as a class of metonymy.

^3^We distinguish in this case between the results of cognitive processes (in this case logic) and the analysis of cognitive processes in the next section. All language is the result of a cognitive method so all methods could be considered to some degree cognitive.

^4^Definitions from English WordNet.

^5^SUMO can be browsed online at https://www.ontologyportal.org/

^6^We note the DOLCE-WordNet mapping is incomplete so we are inferring the corresponding top-level categories. For SUMO, there is already a formal mapping of all WordNet senses.

^7^In the “10 Things I Hate About You” film (1999), the character Chastity Church asks, “I know you can be underwhelmed and you can be overwhelmed, but can you ever just be whelmed?”

^8^We do not believe that other dictionaries are more principled, but are simply less widely adopted in these kinds of works.

^9^ https://www.wiktionary.org/

^10^Six of the first 100 matches were actually nouns.

^11^We speculate this may be due to the mythological and religious connotations of this word or perhaps due to the practice of making shapes in snow or even a Polish pastry.

^12^As an example, Wiktionary defines “fish” as both “an easy victim for swindling” and “a bad poker player.” The verb is also defined with several very similar meanings including “to hunt fish,” “to search (a body of water) for something other than fish,” “to use as bait when fishing,” and “To (attempt to) find or get hold of an object by searching among other objects”.

## References

[B1] Ahmadi S. McCrae J. P. (2021). Monolingual word sense alignment as a classification problem, in Proceedings of the 11th Global Wordnet Conference, GWC 2021, eds Bosch S. Fellbaum C. Griesel M. Rademaker A. Vossen P. (Potchefstroom: University of South Africa (UNISA); Global Wordnet Association), 73–80.

[B2] Ahmadi S. McCrae J. P. Nimb S. Khan F. Monachini M. Pedersen B. S.. (2020). A multilingual evaluation dataset for monolingual word sense alignment, in Proceedings of The 12th Language Resources and Evaluation Conference, LREC 2020, eds Calzolari N. Béchet F. Blache P. Choukri K. Cieri C. Declerck T. Goggi S. Isahara H. Maegaard B. Mariani J. Mazo H. Moreno A. Odijk J. Piperidis S. (Marseille: European Language Resources Association), 3232–3242.

[B3] Apresjan J. D. (1974). Regular polysemy. Linguistics 142, 5–32. 10.1515/ling.1974.12.142.5

[B4] Banarescu L. Bonial C. Cai S. Georgescu M. Griffitt K. Hermjakob U.. (2013). Abstract meaning representation for sembanking, in Proceedings of the 7th Linguistic Annotation Workshop and Interoperability with Discourse (Sofia: Association for Computational Linguistics), 178–186.

[B5] Bansal M. DeNero J. Lin D. (2012). Unsupervised translation sense clustering, in Proceedings of the 2012 Conference of the North American Chapter of the Association for Computational Linguistics: Human Language Technologies (Montréal, QC: Association for Computational Linguistics), 773–782.

[B6] Bevilacqua M. Maru M. Navigli R. (2020). Generationary or ‘how we went beyond word sense inventories and learned to gloss?, in Proceedings of the 2020 Conference on Empirical Methods in Natural Language Processing, EMNLP 2020, eds Webber B. Cohn T. He Y. Liu Y. (Association for Computational Linguistics), 7207–7221. 10.18653/v1/2020.emnlp-main.585

[B7] Blloshmi R. Tripodi R. Navigli R. (2020). XL-AMR: enabling cross-lingual AMR parsing with transfer learning techniques, in Proceedings of the 2020 Conference on Empirical Methods in Natural Language Processing (EMNLP) (Association for Computational Linguistics), 2487–2500. 10.18653/v1/2020.emnlp-main.195

[B8] Bond F. Vossen P. McCrae J. P. Fellbaum C. (2016). CILI: the collaborative interlingual index, in Proceedings of the 8th Global WordNet Conference, GWC 2016, eds Fellbaum C. Vossen P. Mititelu V. B. Forascu C. (Bucharest: Global Wordnet Association), 50–57.

[B9] Branco A. António Rodrigues J. Salawa M. Branco R. Saedi C. (2020). Comparative probing of lexical semantics theories for cognitive plausibility and technological usefulness, in Proceedings of the 28th International Conference on Computational Linguistics (Barcelona: International Committee on Computational Linguistics), 4004–4019. 10.18653/v1/2020.coling-main.354

[B10] Buitelaar P. (1998). CoreLex: systematic polysemy and underspecification (Ph.D. thesis). Brandeis University, Waltham, MA, United States.

[B11] Copland D. A. de Zubicaray G. I. McMahon K. Eastburn M. (2007). Neural correlates of semantic priming for ambiguous words: an event-related fMRI study. Brain Res. 1131, 163–172. 10.1016/j.brainres.2006.11.016 17173868

[B12] Curtis J. Cabral J. Baxter D. (2006). On the application of the Cyc ontology to word sense disambiguation, in Proceedings of the Nineteenth International Florida Artificial Intelligence Research Society Conference, eds Sutcliffe G. Goebel R. (Melbourne Beach, FL: AAAI Press), 652–657.

[B13] Cuyckens H. Zawada B. E. (2001). Polysemy in cognitive linguistics: selected papers, in International Cognitive Linguistics Conference, Vol. 177 (Amsterdam: John Benjamins Publishing). 10.1075/cilt.177

[B14] Dandala B. Hokamp C. Mihalcea R. Bunescu R. C. (2013). Sense clustering using Wikipedia, in Recent Advances in Natural Language Processing, RANLP 2013 (Hissar: RANLP 2013 Organising Committee; ACL), 164–171.

[B15] De Deyne S. Navarro D. J. Perfors A. Brysbaert M. Storms G. (2019). The “small world of words” English word association norms for over 12,000 cue words. Behav. Res. Methods 51, 987–1006. 10.3758/s13428-018-1115-7 30298265

[B16] Devlin J. Chang M. Lee K. Toutanova K. (2019). BERT: pre-training of deep bidirectional transformers for language understanding, in Proceedings of the 2019 Conference of the North American Chapter of the Association for Computational Linguistics: Human Language Technologies, NAACL-HLT 2019, eds Burstein J. Doran C. Solorio T. (Minneapolis, MN: Association for Computational Linguistics), 4171–4186.

[B17] Diab M. Resnik P. (2002). An unsupervised method for word sense tagging using parallel corpora, in Proceedings of the 40th Annual Meeting of the Association for Computational Linguistics (Philadelphia, PA: Association for Computational Linguistics), 255–262. 10.3115/1073083.1073126

[B18] Fellbaum C. (2010). WordNet, in Theory and Applications of Ontology: Computer Applications, eds Ploi R. Healy M. Kameas A. eds (Dordrecht: Springer), 231–243. 10.1007/978-90-481-8847-5_10

[B19] Fillmore C. J. Atkins B. T. (2000). Describing polysemy: the case of 'crawl'. Polysemy 91:110. Available online at: https://global.oup.com/academic/product/polysemy-9780198238423?cc=us&lang=en&#

[B20] Firth J. (1957). A synopsis of linguistic theory 1930-1955, in Studies in Linguistic Analysis: 1–32, ed Palmer F. R. (Longman). Available online at: https://www.bibsonomy.org/bibtex/20b627387b63b652898cb5ecf03f87356/evabl444

[B21] Forcada M. L. Ginestí-Rosell M. Nordfalk J. O'Regan J. Ortiz-Rojas S. Pérez-Ortiz J. A.. (2011). Apertium: a free/open-source platform for rule-based machine translation. Mach. Transl. 25, 127–144. 10.1007/s10590-011-9090-0

[B22] Fortunato S. (2010). Community detection in graphs. Phys. Rep. 486, 75–174. 10.1016/j.physrep.2009.11.002

[B23] Gale W. A. Church K. W. Yarowsky D. (1992). One sense per discourse, in Speech and Natural Language: Proceedings of a Workshop Held at Harriman (New York, NY). 10.3115/1075527.1075579

[B24] Gangemi A. Guarino N. Masolo C. Oltramari A. Schneider L. (2002). Sweetening ontologies with DOLCE, in International Conference on Knowledge Engineering and Knowledge Management (Sigüenza: Springer), 166–181. 10.1007/3-540-45810-7_18

[B25] Gärdenfors P. (2004). Conceptual Spaces: The Geometry of Thought. Cambridge, MA: MIT Press.

[B26] Gibbs R. W. (1999). Researching metaphor, in Researching and Applying Metaphor, eds Cameron L. Low G. (Cambridge: Cambridge University Press), 29–47. 10.1017/CBO9781139524704.005

[B27] Girvan M. Newman M. E. (2002). Community structure in social and biological networks. Proc. Natl. Acad. Sci. U.S.A. 99, 7821–7826. 10.1073/pnas.122653799 12060727 PMC122977

[B28] Goddard C Wierzbicka A. (2013). Words and Meanings: Lexical Semantics Across Domains, Languages, and Cultures. Oxford: Oxford University Press. 10.1093/acprof:oso/9780199668434.001.0001

[B29] Goddard C. (2000). Polysemy: a problem of definition, in Polysemy: Theoretical and Computational Approaches (Oxford: Oxford University Press), 129–151.

[B30] Goel S. Gracia J. Forcada M. L. (2021). Bilingual dictionary generation and enrichment via graph exploration. Semant. Web J. Available online at: http://www.semantic-web-journal.net/content/bilingual-dictionary-generation-and-enrichment-graph-exploration-0

[B31] Gómez-Pérez J. M. Pan J. Z. Vetere G. Wu H. (2017). Enterprise knowledge graph: an introduction, in Exploiting Linked Data and Knowledge Graphs in Large Organisations, eds Pan J. Z. Vetere G. Gómez-Pérez J. M. Wu H. (Cham: Springer), 1–14. 10.1007/978-3-319-45654-6_1

[B32] Gracia J. Montiel-Ponsoda E. Cimiano P. Gómez-Pérez A. Buitelaar P. McCrae J. P. (2012). Challenges for the multilingual web of data. J. Web Semant. 11, 63–71. 10.1016/j.websem.2011.09.001

[B33] Gracia J. Villegas M. Gomez-Perez A. Bel N. (2018). The Apertium bilingual dictionaries on the web of data. Semant. Web 9, 231–240. 10.3233/SW-170258

[B34] Hartmann R. R. K. James G. (1998). Dictionary of Lexicography. London: Routledge. 10.4324/9780203159040

[B35] Hauer B. Kondrak G. (2020). One homonym per translation, in The Thirty-Fourth AAAI Conference on Artificial Intelligence, AAAI 2020, The Thirty-Second Innovative Applications of Artificial Intelligence Conference, IAAI 2020, The Tenth AAAI Symposium on Educational Advances in Artificial Intelligence, EAAI 2020 (New York, NY: AAAI Press), 7895–7902. 10.1609/aaai.v34i05.6296

[B36] Hino Y. Pexman P. M. Lupker S. J. (2006). Ambiguity and relatedness effects in semantic tasks: are they due to semantic coding? J. Mem. Lang. 55, 247–273. 10.1016/j.jml.2006.04.001

[B37] Hovy E. Marcus M. Palmer M. Ramshaw L. Weischedel R. (2006). OntoNotes: the 90% solution, in Proceedings of the Human Language Technology Conference of the NAACL, (New York, NY: Association for Computational Linguistics), 57–60. 10.3115/1614049.1614064

[B38] Jakubíček M. Kilgarriff A. Kovář V. Rychlý P. Suchomel V. (2013). The TenTen corpus family, in 7th International Corpus Linguistics Conference CL (Lancaster: Corpus Linguistics), 125–127.

[B39] Kilgarriff A. (1997). I don't believe in word senses. Comput. Human. 31, 91–113. 10.1023/A:10005839110915302595

[B40] Kilgarriff A. Baisa V. Bušta J. Jakubíček M. Kovář V. Michelfeit J.. (2014). The sketch engine: ten years on. Lexicography 1, 7–36. 10.1007/s40607-014-0009-9

[B41] Kocoń J. Maziarz M. (2021). Mapping WordNet onto human brain connectome in emotion processing and semantic similarity recognition. Inform. Process. Manage. 58:102530. 10.1016/j.ipm.2021.102530

[B42] Krek S. Declerck T. McCrae J. P. Wissik T. (2019). Towards a global lexicographic infrastructure, in Proceedings of the Language Technology for All Conference (Paris).

[B43] Krek S. McCrae J. Kosem I. Wissek T. Tiberius C. Navigli R.. (2018). European Lexicographic Infrastructure (ELEXIS), in Proceedings of the XVIII EURALEX International Congress on Lexicography in Global Contexts (Ljubljana), 881–892.

[B44] Lake B. M. Murphy G. L. (2020). Word meaning in minds and machines. CoRR abs/2008.01766. 10.1037/rev0000297 34292021

[B45] Levine Y. Lenz B. Dagan O. Ram O. Padnos D. Sharir O.. (2020). Sense bert: driving some sense into BERT, in Proceedings of the 58th Annual Meeting of the Association for Computational Linguistics, ACL 2020, eds Jurafsky D. Chai J. Schluter N. Tetreault J. R. (Association for Computational Linguistics), 4656–4667. 10.18653/v1/2020.acl-main.42320011810

[B46] Loureiro D. Jorge A. (2019). Language modelling makes sense: propagating representations through WordNet for full-coverage word sense disambiguation, in Proceedings of the 57th Conference of the Association for Computational Linguistics, ACL 2019, eds Korhonen A. Traum D. R. Márquez L. (Florence: Association for Computational Linguistics.), 5682–5691. 10.18653/v1/P19-1569

[B47] Lyons J. John L. (1995). Linguistic Semantics: An Introduction. Cambridge: Cambridge University Press. 10.1017/CBO9780511810213

[B48] McCrae J. P. Rademaker A. Bond F. Rudnicka E. Fellbaum C. (2019). English WordNet 2019 - an open-source WordNet for English, in Proceedings of the 10th Global Wordnet Conference, GWC 2019, eds Vossen P. Fellbaum C. (Wroclaw: Global Wordnet Association), 245–252.

[B49] McGuinness D. L. Van Harmelen F. (2004). OWL Web Ontology Language Overview. W3C Recommendation. World Wide Web Consortium.

[B50] McShane M. Nirenburg S. Beale S. (2005). An NLP lexicon as a largely language-independent resource. Mach. Transl. 19, 139–173. 10.1007/s10590-006-9001-y

[B51] Měchura M. B. (2017). Introducing Lexonomy: an open-source dictionary writing and publishing system, in Electronic Lexicography in the 21st Century: Lexicography From Scratch. Proceedings of the eLex 2017 Conference, (Leiden), 19–21.

[B52] Mikolov T. Chen K. Corrado G. Dean J. (2013). Efficient estimation of word representations in vector space, in 1st International Conference on Learning Representations, ICLR 2013, eds Bengio Y. LeCun Y. (Scottsdale, AZ).

[B53] Mikolov T. Grave E. Bojanowski P. Puhrsch C. Joulin A. (2018). Advances in pre-training distributed word representations, in Proceedings of the Eleventh International Conference on Language Resources and Evaluation, LREC 2018, eds Calzolari N. Choukri K. Cieri C. Declerck T. Goggi S. Hasida K. Isahara H. Maegaard B. Mariani J. Mazo H. Moreno A. Odijk J. Piperidis S. Tokunaga T. (Miyazaki: European Language Resources Association).

[B54] Miller G. A. (1995). WordNet: a lexical database for English. Commun. ACM 38, 39–41. 10.1145/219717.219748

[B55] Nair S. Srinivasan M. Meylan S. (2020). Contextualized word embeddings encode aspects of human-like word sense knowledge, in Proceedings of the Workshop on the Cognitive Aspects of the Lexicon (Barcelona: Association for Computational Linguistics), 129–141.

[B56] Navigli R. (2006). Meaningful clustering of senses helps boost word sense disambiguation performance, in ACL 2006, 21st International Conference on Computational Linguistics and 44th Annual Meeting of the Association for Computational Linguistics, eds Calzolari N. Cardie C. Isabelle P. (Sydney, NSW: The Association for Computer Linguistics). 10.3115/1220175.1220189

[B57] Navigli R. Ponzetto S. P. (2012). BabelNet: the automatic construction, evaluation and application of a wide-coverage multilingual semantic network. Artif. Intell. 193, 217–250. 10.1016/j.artint.2012.07.001

[B58] Nelson J. S. Grande T. C. Wilson M. V. (2016). Fishes of the World. John Wiley & Sons. 10.1002/9781119174844

[B59] Niles I. Pease A. (2001). Towards a standard upper ontology, in 2nd International Conference on Formal Ontology in Information Systems, FOIS 2001 (Ogunquit, ME), 2–9. 10.1145/505168.505170

[B60] Niles I. Pease A. (2003). Linking lexicons and ontologies: mapping wordnet to the suggested upper merged ontology, in Proceedings of the 2003 International Conference on Information and Knowledge Engineering (Ike 03) (LAS VEGAS), 412–416.

[B61] Nunberg G. (1987). Poetic and prosaic metaphors, in Theoretical Issues in Natural Language Processing 3, TINLAP 1987, ed Wilks Y. (Las Cruces: Association for Computational Linguistics). 10.3115/980304.980349

[B62] Nunberg G. (1992). Systematic polysemy in lexicology and lexicography, in Proceedings of the 5th EURALEX International Congress, eds Tommola H. Varantola K. (Tampere: Tampereen YIiopisto), 386–396.

[B63] Panchenko A. Ruppert E. Faralli S. Ponzetto S. P. Biemann C. (2017). Unsupervised does not mean uninterpretable: the case for word sense induction and disambiguation, in Proceedings of the 15th Conference of the European Chapter of the Association for Computational Linguistics, EACL 2017, eds Lapata M. Blunsom P. Koller A. (Valencia: Association for Computational Linguistics), 86–98. 10.18653/v1/E17-1009

[B64] Pereira B. Robin C. Daudert T. McCrae J. P. Mohanty P. Buitelaar P. (2019). Taxonomy extraction for customer service knowledge base construction, in Semantic Systems. The Power of AI and Knowledge Graphs - 15th International Conference, SEMANTiCS 2019, Vol. 11702 of Lecture Notes in Computer Science, eds Acosta M. Cudré-Mauroux P. Maleshkova M. Pellegrini T. Sack H. Sure-Vetter Y. (Karlsruhe: Springer), 175–190. 10.1007/978-3-030-33220-4_13

[B65] Prokofyev R. Demartini G. Boyarsky A. Ruchayskiy O. Cudré-Mauroux P. (2013). Ontology-based word sense disambiguation for scientific literature, in European Conference on Information Retrieval (Moscow: Springer), 594–605. 10.1007/978-3-642-36973-5_50

[B66] Pustejovsky J. (1991). The generative lexicon. Comput. Linguist. 17, 409–441.

[B67] Pustejovsky J. (1998). The Generative Lexicon. Cambridge, MA: MIT Press. 10.7551/mitpress/3225.001.0001

[B68] Pustejovsky J. Bouillon P. (1995). Aspectual coercion and logical polysemy. J. Semant. 12, 133–162. 10.1093/jos/12.2.133

[B69] Pustejovsky J. Jezek E. (2008). Semantic coercion in language: beyond distributional analysis. Ital. J. Linguist. 20, 175–208. Available online at: https://www.italian-journal-linguistics.com/2008-2/

[B70] Reyes-Maga na J. Sierra Martínez G. Bel-Enguix G. Gomez-Adorno H. (2020). Automatic word association norms (AWAN), in Proceedings of the Workshop on the Cognitive Aspects of the Lexicon (Barcelona: Association for Computational Linguistics), 142–153.

[B71] Rothe S. Schütze H. (2015). AutoExtend: extending word embeddings to embeddings for synsets and lexemes, in Proceedings of the 53rd Annual Meeting of the Association for Computational Linguistics and the 7th International Joint Conference on Natural Language Processing of the Asian Federation of Natural Language Processing, ACL 2015 (Beijing: The Association for Computer Linguistics), 1793–1803. 10.3115/v1/P15-1173

[B72] Sachs O. Weis S. Zellagui N. Sass K. Huber W. Zvyagintsev M.. (2011). How different types of conceptual relations modulate brain activation during semantic priming. J. Cogn. Neurosci. 23, 1263–1273. 10.1162/jocn.2010.21483 20350178

[B73] Scarlini B. Pasini T. Navigli R. (2020). SensEmBERT: context-enhanced sense embeddings for multilingual word sense disambiguation, in The Thirty-Fourth AAAI Conference on Artificial Intelligence, AAAI 2020, The Thirty-Second Innovative Applications of Artificial Intelligence Conference, IAAI 2020, The Tenth AAAI Symposium on Educational Advances in Artificial Intelligence, EAAI 2020 (New York, NY: AAAI Press), 8758–8765. 10.1609/aaai.v34i05.6402

[B74] Shutova E. (2015). Design and evaluation of metaphor processing systems. Comput. Linguist. 41, 579–623. 10.1162/COLI_a_00233

[B75] Snow R. Prakash S. Jurafsky D. Ng A. Y. (2007). Learning to merge word senses, in EMNLP-CoNLL 2007, Proceedings of the 2007 Joint Conference on Empirical Methods in Natural Language Processing and Computational Natural Language Learning, ed Eisner J. (Prague: ACL), 1005–1014.

[B76] Snyder B. Palmer M. (2004). The English all-words task, in Proceedings of SENSEVAL-3, the Third International Workshop on the Evaluation of Systems for the Semantic Analysis of Text (Barcelona: Association for Computational Linguistics), 41–43.

[B77] Szalay L. B. Deese J. (1978). Subjective Meaning and Culture: An Assessment Through Word Associations. Hillsdale, NJ: Lawrence Erlbaum Associates.

[B78] Taghipour K. Ng H. T. (2015). One million sense-tagged instances for word sense disambiguation and induction” in Proceedings of the Nineteenth Conference on Computational Natural Language Learning (Beijing: Association for Computational Linguistics), 338–344. 10.18653/v1/K15-1037

[B79] Van der Maaten L. Hinton G. (2008). Visualizing data using t-SNE. J. Mach. Learn. Res. 9, 2579–2605. Available online at: https://www.jmlr.org/papers/v9/vandermaaten08a.html

[B80] Vicente A. (2018). Polysemy and word meaning: an account of lexical meaning for different kinds of content words. Philos. Stud. 175, 947–968. 10.1007/s11098-017-0900-y

[B81] Walter E. (2010). Using corpora to write dictionaries, in The Routledge Handbook of Corpus Linguistics, ed O'Keeffe A. McCarthy M. (London: Routledge Abingdon-on-Thames), 428–443. 10.4324/9780203856949-31

[B82] Wang A. Pruksachatkun Y. Nangia N. Singh A. Michael J. Hill F.. (2019). SuperGLUE: a stickier benchmark for general-purpose language understanding systems, in Advances in Neural Information Processing Systems 32: Annual Conference on Neural Information Processing Systems 2019, NeurIPS 2019, eds Wallach H. M. Larochelle H. Beygelzimer A. d'Alché-Buc F. Fox B. Garnett R. (Vancouver, BC), 3261–3275.

[B83] Westera M. Boleda G. (2019). Don't blame distributional semantics if it can't do entailment, in Proceedings of the 13th International Conference on Computational Semantics, IWCS 2019, eds Dobnik S. Chatzikyriakidis S. Demberg V. (Gothenburg: Association for Computational Linguistics), 120–133. 10.18653/v1/W19-0410

[B84] Zayed O. McCrae J. P. Buitelaar P. (2020a). Contextual modulation for relation-level metaphor identification, in Findings of the Association for Computational Linguistics: EMNLP 2020. 10.18653/v1/2020.findings-emnlp.36

[B85] Zayed O. McCrae J. P. Buitelaar P. (2020b). Figure me out: a gold standard dataset for metaphor interpretation, in Proceedings of the 12th Language Resource and Evaluation Conference (Marseille), 5810–5819.

